# Correction: Self-report assessment of Positive Appraisal Style (PAS): Development of a process-focused and a content-focused questionnaire for use in mental health and resilience research

**DOI:** 10.1371/journal.pone.0338242

**Published:** 2025-12-05

**Authors:** Papoula Petri-Romão, Haakon Engen, Anna Rupanova, Lara Puhlmann, Matthias Zerban, Rebecca J. Neumann, Aliaksandr Malyshau, Kira F. Ahrens, Anita Schick, Bianca Kollmann, Michèle Wessa, Henrik Walter, Michael M. Plichta, Andreas Reif, Andrea Chmitorz, Oliver Tuescher, Ulrike Basten, Raffael Kalisch

The 12^th^ author’s name is spelled incorrectly. The correct name is: Henrik Walter

S5 Table was uploaded incorrectly. Please see the correct S5 Table here.

## Supporting information

S5 Table PASS-process German version. (DOCX)
