# Supplementary figures and images for: Self-report assessment of Positive Appraisal Style (PAS): Development of a process-focused and a content-focused questionnaire for use in mental health and resilience research
Source: PLoS One. 2024 Feb 2;19(2):e0295562. doi: 10.1371/journal.pone.0295562 (PMC10836662; doi:10.1371/journal.pone.0295562)

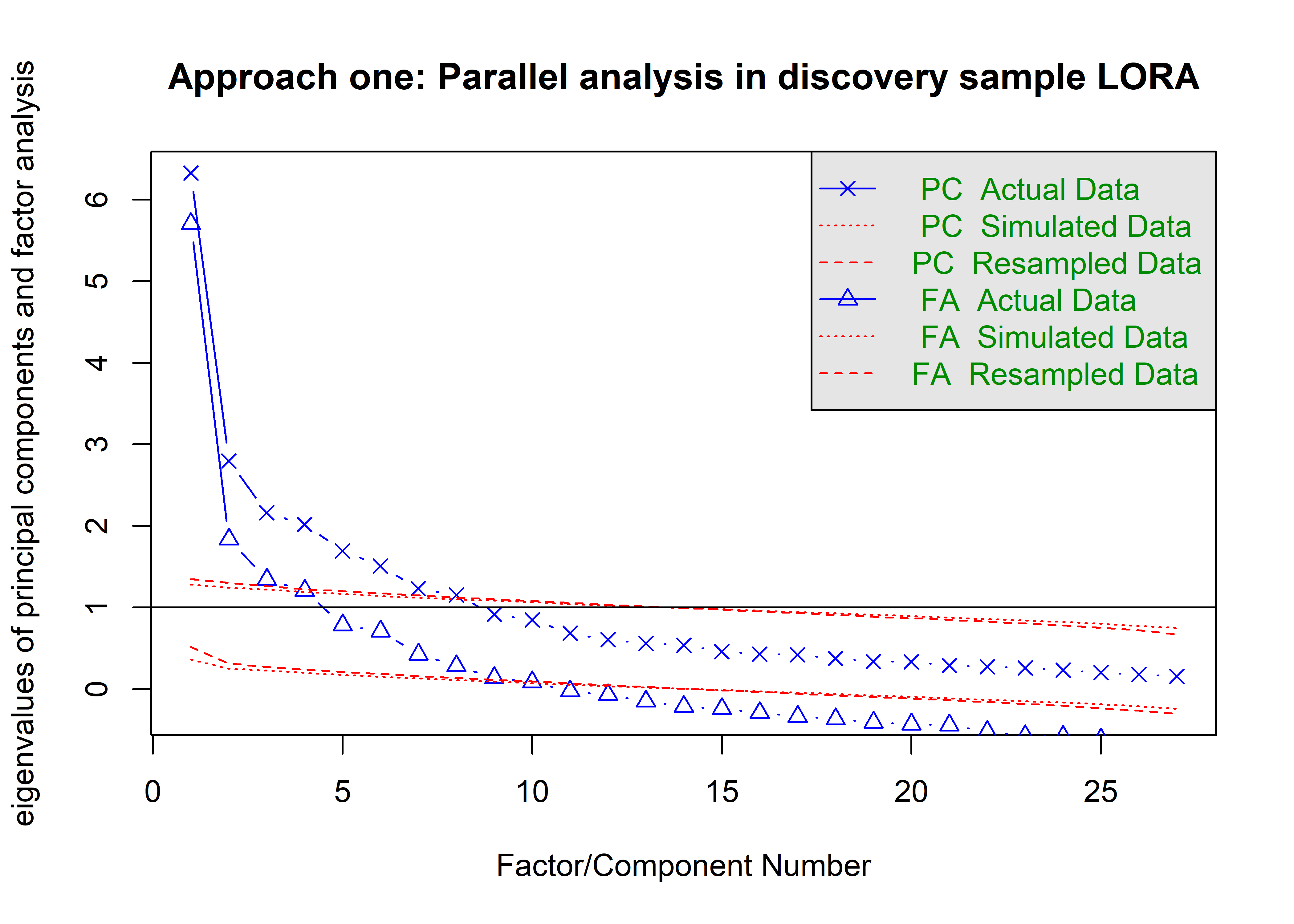

Supplement: S1 Fig — (TIF) [file pone.0295562.s001.tif]

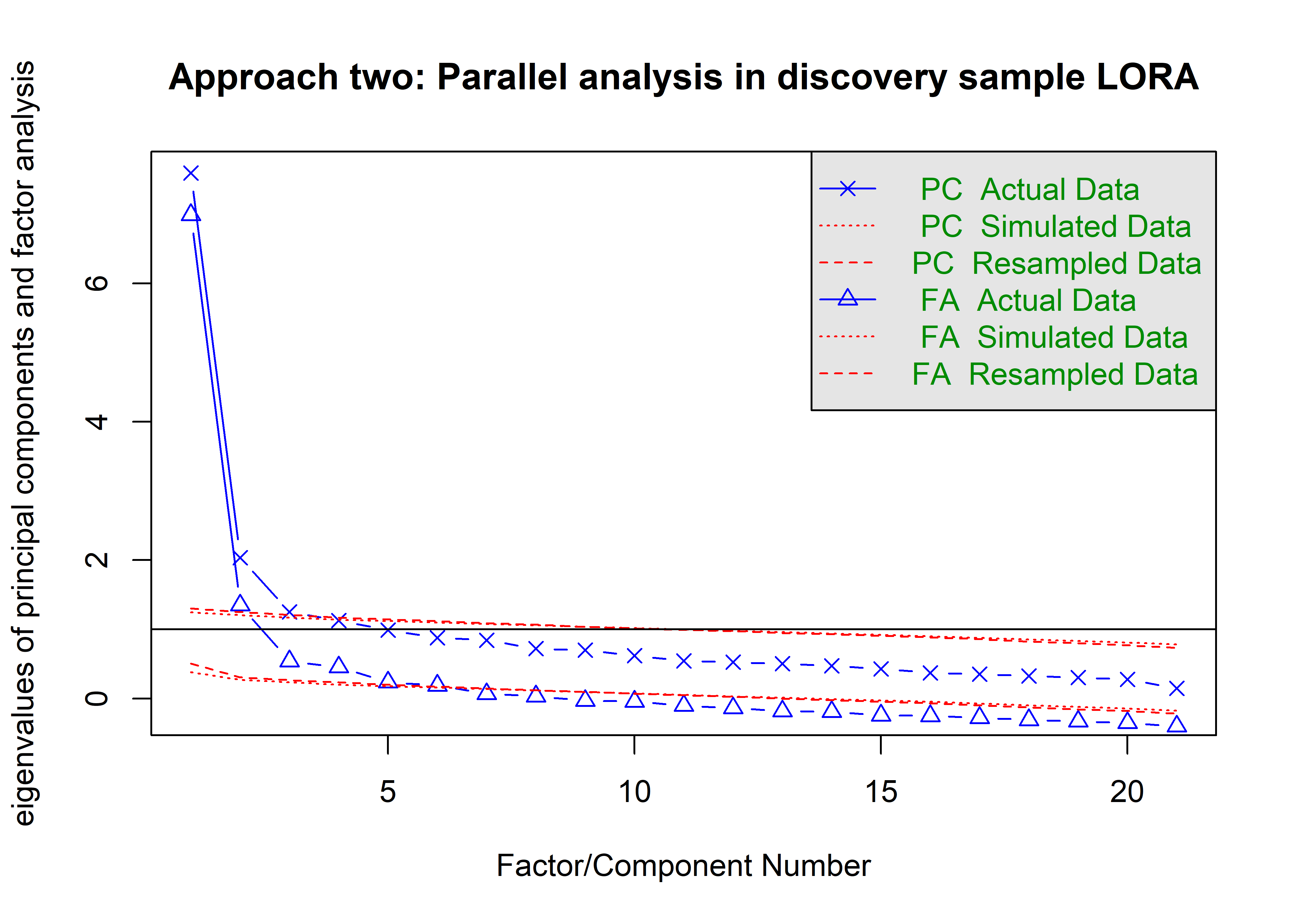

Supplement: S2 Fig — (TIF) [file pone.0295562.s002.tif]
