# Supplementary material for: Self-report assessment of Positive Appraisal Style (PAS): Development of a process-focused and a content-focused questionnaire for use in mental health and resilience research
Source: PLoS One. 2024 Feb 2;19(2):e0295562. doi: 10.1371/journal.pone.0295562 (PMC10836662; doi:10.1371/journal.pone.0295562)
Supplement: S1 Table — (DOCX) [file pone.0295562.s003.docx]

## Table S1. Overview of subscales in the Brief COPE and CERQ-short and self-generated Distancing subscale.

| Number of Subscales | Name of subscales | Example item | Inclusion |
| --- | --- | --- | --- |
| Brief COPE | | | |
| 14 | Self-distraction | I have been turning to work or other activities to take my mind off things. | Excluded |
|  | Active coping | I have been concentrating my efforts on doing something about the situation I'm in. | Excluded |
|  | Denial | I have been saying to myself "this isn't real." | Excluded |
|  | Substance use | I have been using alcohol or other drugs to make myself feel better. | Excluded |
|  | Use of emotional support | I have been getting emotional support from others. | Excluded |
|  | Use of instrumental support | I have been getting help and advice from other people. | Excluded |
|  | Behavioral disengagement | I have been giving up trying to deal with it. | **Included** |
|  | Venting | I have been saying things to let my unpleasant feelings escape. | Excluded |
|  | Positive reframing | I have been trying to see it in a different light, to make it seem more positive. | **Included** |
|  | Planning | I have been trying to come up with a strategy about what to do. | Excluded |
|  | Humor | I have been making jokes about it. | **Included** |
|  | Acceptance | I have been accepting the reality of the fact that it has happened. | **Included** |
|  | Turning to Religion | I have been praying or meditating.” | **Included** |
|  | Self-blame | I have been blaming myself for things that happened. | Excluded |
| CERQ-short | | | |
| 7 | Self-blame | “I feel that I am the one who is responsible for what has happened.” | Excluded |
|  | Acceptance | “I think that I have to accept that this has happened.” | **Included** |
|  | Rumination | “I often think about how I feel about what I have experienced.” | **Included** |
|  | Positive refocusing | “I think of pleasant things that have nothing to do with it.” | Excluded |
|  | Refocus on planning | “I think about a plan of what I can do best.” | Excluded |
|  | Positive reappraisal | “I think I can learn something from the situation.” | **Included** |
|  | Putting into perspective | “I think that it hasn’t been too bad compared to other things.” | **Included** |
|  | Catastrophizing | “I keep thinking about how terrible it is what I have experienced.” | Excluded |
|  | Other-blame | “I feel that others are responsible for what has happened.” | **Included** |
| Self-generated | | | |
| 1 | Distancing | “I try to look at the situation from an objective perspective.” | **Included** |
