# Supplementary material for: Self-report assessment of Positive Appraisal Style (PAS): Development of a process-focused and a content-focused questionnaire for use in mental health and resilience research
Source: PLoS One. 2024 Feb 2;19(2):e0295562. doi: 10.1371/journal.pone.0295562 (PMC10836662; doi:10.1371/journal.pone.0295562)
Supplement: S2 Table — (DOCX) [file pone.0295562.s004.docx]

**Table S2. CERQ item Evaluation.**

|  | Question | Subscale | kurtosis | skewness | difficulty | missing | mean | SD |
| --- | --- | --- | --- | --- | --- | --- | --- | --- |
| cerq2 | I think that I have to accept that this has happened | Acceptance | 2.02 | -0.27 | 0.71 | 0.25% | 3.53 | 1.12 |
| cerq3 | I often think about how I feel about what I have experienced | Rumination | 1.89 | 0.04 | 0.63 | 0.25% | 3.15 | 1.21 |
| cerq6 | I think that I can become a stronger person as a result of what has happened | Positive Reappraisal | 2.14 | -0.5 | 0.72 | 0.25% | 3.61 | 1.21 |
| cerq7 | I think that other people go through much worse experiences | Putting into Perspective | 2.24 | -0.59 | 0.74 | 0.34% | 3.69 | 1.2 |
| cerq9 | I feel that others are to blame for it | Other-blame | 5.45 | 1.27 | 0.36 | 0.25% | 1.8 | 0.79 |
| cerq11 | I think that I have to accept the situation | Acceptance | 2.05 | -0.17 | 0.69 | 0.25% | 3.46 | 1.11 |
| cerq12 | I am preoccupied with what I think and feel about what I have experienced | Rumination | 3.04 | 0.83 | 0.43 | 0.25% | 2.15 | 1.04 |
| cerq15 | I look for the positive sides to the matter | Positive Reappraisal | 2.01 | -0.1 | 0.65 | 0.42% | 3.27 | 1.16 |
| cerq16 | I think that it hasn’t been too bad compared to other things | Putting into Perspective | 2.07 | 0.02 | 0.63 | 0.25% | 3.16 | 1.12 |
| cerq18 | I feel that others are responsible for what has happened | Other-blame | 5.45 | 1.22 | 0.37 | 0.25% | 1.85 | 0.79 |
| cerq20 | I think that I must learn to live with it | Acceptance | 2.1 | -0.09 | 0.65 | 0.25% | 3.27 | 1.13 |
| cerq21 | I want to understand why I feel the way I do about what I have experienced | Rumination | 1.79 | 0.03 | 0.6 | 0.25% | 2.98 | 1.33 |
| cerq24 | I look for the positive sides to the matter | Positive Reappraisal | 2.02 | -0.19 | 0.67 | 0.25% | 3.36 | 1.17 |
| cerq25 | I think that it hasn’t been too bad compared to other things I tell myself that there are worse things in life | Putting into Perspective | 2.1 | -0.39 | 0.72 | 0.34% | 3.6 | 1.15 |
| cerq27 | I feel that basically the cause lies with others | Other-blame | 6.3 | 1.66 | 0.31 | 0.25% | 1.54 | 0.77 |
| cerq28 | I try to look at the situation from an objective perspective. | Distancing | 1.99 | -0.04 | 0.62 | 1.85% | 3.12 | 1.23 |
| cerq29 | I try to distance myself from the situation and my feelings. | Distancing | 2.42 | 0.45 | 0.52 | 1.68% | 2.6 | 1.13 |
