# Supplementary material for: Self-report assessment of Positive Appraisal Style (PAS): Development of a process-focused and a content-focused questionnaire for use in mental health and resilience research
Source: PLoS One. 2024 Feb 2;19(2):e0295562. doi: 10.1371/journal.pone.0295562 (PMC10836662; doi:10.1371/journal.pone.0295562)
Supplement: S3 Table — (DOCX) [file pone.0295562.s005.docx]

## Table S3. COPE item evaluation.

|  | Question | Subscale | kurtosis | skewness | difficulty | missing | mean | SD |
| --- | --- | --- | --- | --- | --- | --- | --- | --- |
| cope6 | I have been giving up trying to deal with it. | Behavioral disengagement | 3.12 | 0.84 | 0.4 | 0.34% | 1.59 | 0.67 |
| cope12 | I have been trying to see it in a different light, to make it seem more positive | Positive Reframing | 2.35 | -0.16 | 0.7 | 0.42% | 2.8 | 0.83 |
| cope16 | I have been giving up the attempt to cope. | Behavioral disengagement | 10.85 | 2.72 | 0.3 | 0.42% | 1.21 | 0.5 |
| cope17 | I have been looking for something good in what is happening. | Positive Reframing | 2.21 | 0.02 | 0.66 | 0.42% | 2.63 | 0.88 |
| cope18 | I have been making jokes about it. | Humor | 2.38 | 0.46 | 0.53 | 0.34% | 2.14 | 0.93 |
| cope20 | I have been accepting the reality of the fact that it has happened. | Acceptance | 2.37 | -0.09 | 0.66 | 0.42% | 2.66 | 0.85 |
| cope22 | I have been trying to find comfort in my religion or spiritual beliefs. | Religion | 4.08 | 1.46 | 0.39 | 0.34% | 1.57 | 0.89 |
| cope24 | I have been learning to live with it. | Acceptance | 2.28 | -0.14 | 0.72 | 0.34% | 2.89 | 0.79 |
| cope27 | I have been praying or meditating. | Religion | 4.37 | 1.53 | 0.39 | 0.34% | 1.55 | 0.87 |
| cope28 | I have been making fun of the situation. | Humor | 2.73 | 0.51 | 0.53 | 0.34% | 2.12 | 0.85 |
