# Supplementary material for: Self-report assessment of Positive Appraisal Style (PAS): Development of a process-focused and a content-focused questionnaire for use in mental health and resilience research
Source: PLoS One. 2024 Feb 2;19(2):e0295562. doi: 10.1371/journal.pone.0295562 (PMC10836662; doi:10.1371/journal.pone.0295562)
Supplement: S4 Table — (DOCX) [file pone.0295562.s006.docx]

## Table S4. Perceived Positive Appraisal Style Scale, process-focused.

| Item No | Item |
| --- | --- |
| How do you cope with events?  Everyone gets confronted with negative or unpleasant events now and then and everyone responds to them in his or her own way. In the following questions you are asked to indicate what you usually think when you experience negative or unpleasant events. Here we are interested in your general tendencies.  1 = (almost) never, 2 = sometimes, 3= regularly, 4= often 5= (almost) always | |
| PASS-process_01 | I make jokes about it. |
| PASS-process_02 | I make fun of the situation. |
| PASS-process_03 | I think that I have to accept that this has happened. |
| PASS-process_04 | I think that I can become a stronger person as a result of what has happened. |
| PASS-process_05 | I think that I have to accept the situation. |
| PASS-process_06 | I think that the situation also has its positive sides. |
| PASS-process_07 | I think that it hasn’t been too bad compared to other things. |
| PASS-process_08 | I tell myself that there are worse things in life. |
| PASS-process_09 | I try to look at the situation from an objective perspective. |
| PASS-process_10 | I try to distance myself from the situation and my feelings. |
