# Supplementary material for: Self-report assessment of Positive Appraisal Style (PAS): Development of a process-focused and a content-focused questionnaire for use in mental health and resilience research
Source: PLoS One. 2024 Feb 2;19(2):e0295562. doi: 10.1371/journal.pone.0295562 (PMC10836662; doi:10.1371/journal.pone.0295562)
Supplement: S6 Table — (DOCX) [file pone.0295562.s008.docx]

## Table S6. *Evaluation of the items in the item pool for PAS.*

| No. | Item | Categorization | kurtosis | skewness | Item-total correlation (part-whole corrected) | Item difficulty | Missings | Mean | SD |
| --- | --- | --- | --- | --- | --- | --- | --- | --- | --- |
| 1^a^ | Ich sehe die Dinge eher pessimistisch. | Cross-dimensional appraisal | 3.24 | -0.59 | 0.58 | 0.79 | 1.01 % | 3.17 | 0.71 |
| 2^a,x^ | Ich denke mir, Angst und Stress sind sehr schädlich und sollten vermieden werden. | Outcome magnitude appraisal | 2.30 | 0.15 | -0.04 | 0.58 | 1.01 % | 2.34 | 0.88 |
| 3 | Ich denke mir, jede schwierige Situation ist irgendwann auch wieder zu Ende. | Outcome probability appraisal | 3.09 | -0.84 | 0.57 | 0.84 | 1.09 % | 3.36 | 0.72 |
| 4 | Ich denke mir, ich kann selbst mit der schlimmsten Situation fertig werden. | Coping potential appraisal | 2.62 | -0.44 | 0.55 | 0.76 | 1.01 % | 3.03 | 0.79 |
| 5 | Ich denke mir, dass es auch in schlimmen Dingen einen Sinn gibt. | Appraisal Background | 2.18 | -0.07 | 0.47 | 0.68 | 1.09 % | 2.72 | 0.88 |
| 6^a,x^ | Es ist mir vor allem wichtig, mir keine Blöße zu geben und mich nicht lächerlich zu machen. | Appraisal Background | 2.34 | -0.11 | 0.19 | 0.63 | 1.01 % | 2.52 | 0.87 |
| 7 | Ich denke mir, man darf sich von Kleinigkeiten nicht aus dem Konzept bringen lassen | Appraisal Background | 2.91 | -0.53 | 0.46 | 0.77 | 1.01 % | 3.09 | 0.76 |
| 8^a^ | Ich verzweifle schnell. | Coping potential appraisal | 3.66 | -0.84 | 0.50 | 0.83 | 1.01 % | 3.32 | 0.7 |
| 9 | Ich denke mir, wenn man nicht genau weiß, was kommt, ist es besser, ein gutes Ende anzunehmen. | Outcome probability appraisal | 2.34 | -0.30 | 0.48 | 0.71 | 1.09 % | 2.86 | 0.87 |
| 10^a,x^ | Ich denke mir, was klein anfängt, wächst sich oft zu einem großen Problem aus. | Outcome magnitude appraisal | 3.32 | -0.59 | 0.26 | 0.80 | 1.01 % | 3.19 | 0.7 |
| 11^x^ | Ich denke mir, Risiken werden oft überschätzt. | Cross-dimensional appraisal | 3.08 | 0.37 | 0.20 | 0.56 | 1.01 % | 2.22 | 0.73 |
| 12^x^ | Ich denke mir, es gibt Dinge, die wichtiger sind als Erfolg, Anerkennung oder Reichtum. | Appraisal Background | 2.50 | -0.64 | 0.25 | 0.81 | 1.01 % | 3.22 | 0.8 |
| 13^a,x^ | Wenn ich in schwierigen Situationen nervös oder unkonzentriert werde, denke ich mir, dass dies wahrscheinlich Anzeichen für eine psychische Erkrankung sind. | Outcome magnitude appraisal | 8.87 | -2.40 | 0.34 | 0.94 | 1.01 % | 3.75 | 0.55 |
| 14^x^ | Ich habe Vertrauen in Gott / in das Schicksal / in das Leben. | Appraisal Background | 1.73 | 0.20 | 0.21 | 0.57 | 1.01 % | 2.29 | 1.09 |
| 15 | Ich sehe die Dinge eher optimistisch. | Cross-dimensional appraisal | 2.41 | -0.50 | 0.66 | 0.77 | 1.01 % | 3.09 | 0.83 |
| 16^a^ | Ich denke mir, meine Bedürfnisse werden nicht erfüllt. | Cross-dimensional appraisal | 3.10 | -0.46 | 0.40 | 0.81 | 1.01 % | 3.25 | 0.64 |
| 17^a,x^ | Ich versuche, Unannehmlichkeiten möglichst auszuweichen. | Appraisal Background | 2.56 | -0.16 | 0.25 | 0.61 | 1.01 % | 2.45 | 0.77 |
| 18^a^ | Ich nehme eine negative Sichtweise ein. | Appraisal Background | 3.26 | -0.62 | 0.59 | 0.82 | 1.01 % | 3.27 | 0.67 |
| 19 | Ich denke mir, es gibt für alles eine Lösung. | Coping potential appraisal | 3.10 | -0.72 | 0.64 | 0.82 | 1.01 % | 3.27 | 0.73 |
| 20^a^ | Ich traue mir sehr wenig zu. | Coping potential appraisal | 3.27 | -0.87 | 0.45 | 0.84 | 1.01 % | 3.35 | 0.72 |
| 21 | Ich denke mir, wenn man nur durchhält, wird es irgendwann wieder besser. | Outcome probability appraisal | 2.76 | -0.49 | 0.36 | 0.77 | 1.09 % | 3.07 | 0.77 |
| 22 | Ich denke mir, das Leben ist trotz allem wunderbar. | Appraisal Background | 3.14 | -0.90 | 0.52 | 0.85 | 1.01 % | 3.4 | 0.7 |
| 23 | Ich versuche, die Dinge so realistisch zu sehen, wie sie eben sind. | Cross-dimensional appraisal | 2.65 | -0.56 | 0.32 | 0.83 | 1.01 % | 3.34 | 0.66 |
| 24 | Ich denke mir, man sollte aus einer Mücke keinen Elefanten machen. | Outcome magnitude appraisal | 2.44 | -0.45 | 0.41 | 0.78 | 1.01 % | 3.13 | 0.77 |
| 25 | Ich nehme für meine Ziele oder Ideale Unannehmlichkeiten in Kauf. | Appraisal Background | 2.10 | -0.08 | 0.32 | 0.73 | 1.09 % | 2.93 | 0.78 |
| 26^a^ | Ich kann in negativen Erlebnissen nichts Gutes sehen. | Appraisal Background | 3.63 | -0.68 | 0.44 | 0.79 | 1.01 % | 3.17 | 0.71 |
| 27^a^ | Ich denke mir, meine Ziele sind gefährdet. | Cross-dimensional appraisal | 3.50 | -0.75 | 0.39 | 0.83 | 1.01 % | 3.31 | 0.68 |
| 28 | Ich denke mir, irgendwie kriege ich doch immer, was ich brauche. | Cross-dimensional appraisal | 2.74 | -0.36 | 0.47 | 0.74 | 1.01 % | 2.98 | 0.76 |
| 29 | Ich denke mir, dass Dinge, die anfangs schlimm erscheinen, oft doch gut ausgehen. | Outcome magnitude appraisal | 2.58 | -0.17 | 0.54 | 0.74 | 1.01 % | 2.95 | 0.71 |

^a^ Item is negatively worded and reverse coded. ^x^ item was excluded based on item quality indices.
