# Supplementary material for: Self-report assessment of Positive Appraisal Style (PAS): Development of a process-focused and a content-focused questionnaire for use in mental health and resilience research
Source: PLoS One. 2024 Feb 2;19(2):e0295562. doi: 10.1371/journal.pone.0295562 (PMC10836662; doi:10.1371/journal.pone.0295562)
Supplement: S7 Table — (DOCX) [file pone.0295562.s009.docx]

## Table S7. Perceived Positive Appraisal Style Scale, content-focused.

| Item No | Item |
| --- | --- |
| Please think about how you usually act in difficult, uncertain, burdening, stressful or critical situations and what you usually feel and think. Please indicate if the statements below are valid never, sometimes, often or most of the times. There are no right or wrong answers.  1 = never, 2 = sometimes, 3 = often, 4 = almost always | |
| PASS-content_1 | I think that every difficult situation will end eventually. |
| PASS-content_2 | I think that I can deal successfully even with even the worst situation. |
| PASS-content_3 | I think that even bad things have a meaning. |
| PASS-content_4 | I think that you should not be rattled by small things. |
| PASS-content_5 | I think that it is better to assume a good ending if you don’t know what is coming. |
| PASS-content_6 | I tend to see things rather optimistically. |
| PASS-content_7 | I think that there is a solution for every problem. |
| PASS-content_8 | I think that things will get better if you sit through them. |
| PASS-content_9 | I think that life is wonderful after all. |
| PASS-content_10 | I try to see things realistically, like they are. |
| PASS-content_11 | I think that you shouldn’t make mountains out of molehills. |
| PASS-content_12 | For my goals and my ideals, I accept inconvenience. |
| PASS-content_13 | I think that I somehow always manage to get what I need. |
| PASS-content_14 | I think that things that initially seem bad often turn out well in the end. |
