# Supplementary material for: Self-report assessment of Positive Appraisal Style (PAS): Development of a process-focused and a content-focused questionnaire for use in mental health and resilience research
Source: PLoS One. 2024 Feb 2;19(2):e0295562. doi: 10.1371/journal.pone.0295562 (PMC10836662; doi:10.1371/journal.pone.0295562)
Supplement: S8 Table — (DOCX) [file pone.0295562.s010.docx]

## Table S8. PASS-content German Version.

| Number | Item |
| --- | --- |
|  | Typische Reaktionen in schwierigen Situationen. Bitte denken Sie darüber nach, wie Sie sich üblicherweise in schwierigen, unsicheren, belastenden, stressigen oder bedenklichen Situationen verhalten und was Sie üblicherweise fühlen und denken. Geben Sie an, ob die untenstehenden Aussagen nie, manchmal, häufig  oder fast immer zutreffen. Dabei gibt es keine richtigen oder falschen Antworten. 1=nie 2=manchmal 3=häufig 4=fast immer |
| 1 | Ich denke mir, jede schwierige Situation ist irgendwann auch wieder zu Ende. |
| 2 | Ich denke mir, ich kann selbst mit der schlimmsten Situation fertig werden. |
| 3 | Ich denke mir, dass es auch in schlimmen Dingen einen Sinn gibt. |
| 4 | Ich denke mir, man darf sich von Kleinigkeiten nicht aus dem Konzept bringen lassen |
| 5 | Ich denke mir, wenn man nicht genau weiß, was kommt, ist es besser, ein gutes Ende anzunehmen. |
| 6 | Ich sehe die Dinge eher optimistisch. |
| 7 | Ich denke mir, es gibt für alles eine Lösung. |
| 8 | Ich denke mir, wenn man nur durchhält, wird es irgendwann wieder besser. |
| 9 | Ich denke mir, das Leben ist trotz allem wunderbar. |
| 10 | Ich versuche, die Dinge so realistisch zu sehen, wie sie eben sind. |
| 11 | Ich denke mir, man sollte aus einer Mücke keinen Elefanten machen. |
| 12 | Ich nehme für meine Ziele oder Ideale Unannehmlichkeiten in Kauf. |
| 13 | Ich denke mir, irgendwie kriege ich doch immer, was ich brauche. |
| 14 | Ich denke mir, dass Dinge, die anfangs schlimm erscheinen, oft doch gut ausgehen. |
