# Supplementary material for: Correction: Self-report assessment of Positive Appraisal Style (PAS): Development of a process-focused and a content-focused questionnaire for use in mental health and resilience research
Source: PLoS One. 2025 Dec 5;20(12):e0338242. doi: 10.1371/journal.pone.0338242 (PMC12680137; doi:10.1371/journal.pone.0338242)
Supplement: S5 Table — (DOCX) [file pone.0338242.s001.docx]

## Table S5. PASS-process German version.

| No. | Item |
| --- | --- |
| Wie gehen Sie mit Ereignissen um?  Jedem von uns passieren hin und wieder negative oder unangenehme Ereignisse und jeder reagiert darauf auf seine/ihre eigene Art. Für die folgenden Aussagen werden Sie gebeten, anzugeben, was Sie normalerweise denken, wenn Sie negative oder unangenehme Ereignisse erleben. Wir sind hier an Ihren typischen Reaktionen interessiert.  1) (fast) nie  2) manchmal  3) regelmäßig  4) häufig  5) (fast) immer | |
| PASSp_01 | Ich mache Witze darüber. |
| PASSp_02 | Ich nehme alles mit Humor. |
| PASSp_03 | Ich denke, dass ich akzeptieren muss, was passiert ist. |
| PASSp_04 | Ich denke, dass ich aufgrund dessen, was geschehen ist, ein stärkerer Mensch werden kann. |
| PASSp_05 | Ich denke, dass ich die Situation akzeptieren muss. |
| PASSp_06 | Ich denke, die Situation hat auch ihre positiven Seiten. |
| PASSp_07 | Ich denke, dass es gar nicht so schlimm war, im Vergleich zu anderen Dingen. |
| PASSp_08 | Ich sage mir, es gibt schlimmere Dinge im Leben. |
| PASSp_09 | Ich versuche, die Situation aus einer losgelösten Perspektive, wie von außen, zu betrachten. |
| PASSp_10 | Ich versuche, mich von der Situation und meinen Gefühlen zu distanzieren. |
